# Prevalence of cervical neoplastic lesions and Human Papilloma Virus infection in Egypt: National Cervical Cancer Screening Project

**DOI:** 10.1186/1750-9378-2-12

**Published:** 2007-07-04

**Authors:** Howayda S Abd El All, Amany Refaat, Khadiga Dandash

**Affiliations:** 1 Howayda S Abd El All, Principal Investigator for Pathology, Department of Pathology, Faculty of Medicine Suez Canal University, Ismailia, Egypt; 2 Department of Community Medicine, Principal Investigator for Data Management, Faculty of Medicine Suez Canal University, Ismailia, Egypt; 3 Department of Community Medicine, Principal Investigator for Field Work, Faculty of Medicine Suez Canal University, Ismailia, Egypt

## Abstract

**Background:**

Data from Egyptian studies provide widely varying estimates on the prevalence of pre-malignant and malignant cervical abnormalities and human papilloma virus (HPVs) infection. To define the prevalence and risk factors of pre-invasive and invasive cervical cancer (cacx), a community based full-scale cross sectional, household survey including 5453 women aged between 35 and 60 years was conducted.

**Methods:**

The study period was between February 2000 and December 2002. Initially, conventional Papanicolaou (Pap) smears were evaluated using the Bethesda system (TBS), followed by colposcopic guided biopsy (CGB) for all epithelial abnormalities (EA). In a third step, HPV was tested on all EA by in-situ hybridization (ISH) using first the broad spectrum HPV probe recognizing HPVs 6, 11, 16, 18, 30, 31, 35, 45, 51 and 52 followed by subtyping with probes 6/11, 16/18 and 31/33. Lastly, unequivocal cases were immunostained for herpes simplex type-2 (HSV-2), cytomegalovirus (CMV), and human immunodeficiency virus (HIV).

**Results:**

EA representing 7.8% (424/5453), were categorized into atypical squamous cell of undetermined significance (ASCUS) (34.4%), atypical glandular cell of undetermined significance (AGCUS) (15.3%), combined ASCUS and AGCUS (3.1%), low grade squamous intraepithelial lesions (SIL) (41.0%), high grade SIL (5.2%) and invasive lesions (1%). CGB of EA (n = 281) showed non neoplastic lesions (12.8%), atypical squamous metaplasia (ASM) (19.2%), cervical intraepithelial neoplasia I (CIN) (44.4%), CIN II (4.4%), CINIII (2.8%), endocervical lesions (5.2%), combined squamous and endocervical lesions (10.0%), invasive squamous cell carcinoma (SCC) (0.02%) and extranodal marginal zone B cell lymphoma (MZBCL) (0.02%). The overall predictive value of cytology was 87% while the predictive value for high grade lesions was 80%. On histological basis, HPVs were present in 94.3% of squamous lesions while it was difficult to be identified in endocervical ones. ISH revealed positivity for pan HPV in 65.9% of the studied biopsies (n = 217), with incorporation of the viral genome HPV 6/11, 16/18 and 31/33 in 11.1%, 33.3% and 17.1% respectively. Multiple HPVs infections were identified in 0.02%.

**Conclusion:**

Pre-invasive high grade lesions and invasive cervical carcinoma represent 0.5% and 0.04% respectively in Egyptian women. HPV mostly 16/18 as a risk factor (p < 0.001), was frequently associated with mixed infections (p < 0.001) and bilharzial infestation (p < 0.001).

## Background

In developed countries, with effective and extensive screening with Pap smears, it is usually possible to identify and treat asymptomatic precursor lesions of cacx, making it nearly 100% preventable [1]. However, five out of six women with cacx live in developing countries, and 80% of them are diagnosed at advanced stages [2]. Data from Egyptian studies provide widely varying estimates on the prevalence of pre-invasive cervical lesions ranging from 1% [3] to 8% [4] with an age range from 20–60 years. Invasive lesions represented 59.58% of all female genital tract malignancy according to Egyptian National Cancer Institute data [5]. A recent monograph from the Egyptian National Cancer Registry of seven cancer centres of the Ministry of Health and Population, reported an incidence rate for all ages ranging from 0.12% to 0.77% depending on geographical regions, being more prevalent in Lower Egypt [6]. To be stressed here, that most patients presented at late incurable clinical stages [7,8]. Previous small hospital based series confirmed the incorporation of HPV genome in invasive lesions [8,9]. In addition, schistosomiaisis [8,10], and Chlamydia trachomatis (Chlamydia T) [11], has been found to be risk factors in Egyptian studies.

In the present work we looked for HPV and other risk factors [12-17] in the Egyptian population. These include viruses such as HSV-2 [16,18], CMV [16], and HIV [17,19]. Sexual activity with multiple sexual partners, sex with a promiscuous partner, history of sexually transmitted infections, sexual intercourse at a young age [20-22], bacterial vaginosis [23], Chlamydia T [11,24], schistosomiaisis [8,10,25], poor socioeconomic and low educational status [26-29], risky jobs [30] and poor nutritional status [31] have also been implicated as risk factors.

This study was the first one to be conducted in Egypt at a National level as community based full-scale cross sectional survey. The main objective was to assess the prevalence of pre-invasive and invasive cacx among Egyptian women aged between 35 and 60 years. Specifically it aimed at assessing the prevalence of biopsy-confirmed squamous, endocervical lesions and invasive cancer, identifying the preventable risk factors and exploring the knowledge and practices of the target population toward cacx screening. Therefore representative data on this problem should permit better evaluation of possible alternative approaches for the design of a national cacx prevention program.

## Methods

### Duration of the study

The protocol was set up and approved by the funding agency "United States Agency for International Development (USAID) in January 2000. The field work started July 2000 and terminated in December 2002.

### Sampling

#### Sampling design and target population

The prevalence rates used for sample size calculations were chosen based on available data from Egyptian studies and results of recent computer modelling. A computer simulation model of incidence of cacx, based on a low risk population in the United States, suggested that the prevalence of SIL, in a previously unscreened population would be 2–3% among women 35 years old and above. The sample size estimates assume simple random sampling equal to 3012 eligible women. Assuming a design effect of 2 due to cluster sampling, the sample size needs to be doubled to be 6000. The target population was ever-married women aged 35 to 60 years. Women who were pregnant or menstruating at time of tests were excluded.

#### Selection of study population

A multi-stage sampling process was used, based on a sampling frame from the Egypt Demographic Health Survey (DHS) 2000 [32]. A sample of different regions of Egypt was made using data from the Human Development Index, which is a composite index of education, health status and living standard. Ten out of twenty six governorates were randomly chosen for the survey, representing urban/rural regions including Lower Egypt (LE), Upper Egypt (UE) and Urban governorates. From each of the sample governorates, 60 clusters of 230 households were selected that were expected to include 125 eligible women. Allowing for refusals would give about 100 women per cluster.

#### Ethical considerations

The study protocol was approved by the Ministry of Health and Population Ethical Review Board. All women were informed about the procedures of the study, the possibility of abnormalities being found, including cacx and that further invasive procedures may be necessary. Consents were obtained from the women before participation.

### Data collection

#### Questionnaire

A total of 153 questions collected data about women's background and relevant risk factors including 1- socioeconomic and general characteristics of women and their husbands, living conditions, lifestyle, marital status, menstrual and obstetric history and use of family planning, 2- current general health problems with special emphasis on schistosomiaisis, 3- gynecological health problems including personal and family history of cacx or other cancers and women and husbands' history of genital tract infections.

#### Clinical and gynecological examinations

General clinical examinations and urine laboratory test for schistosomiasis by spotting positive ova were carried out. Problems such as cystocoele, rectocoele, adnexal tenderness, or masses, uterine prolapse, cervical polyps, warts and cervical discharge, were noted and evaluated. Pap smears were taken.

### Pathological study

#### Cytopathological examination

Samples from the ectocervix and endocervix were collected using Ayer's spatula and endocervical brush and fixed onto labelled glass slides with cytospray. The cells were classified according to TBS 1991 [33]. Strict criteria were used to qualify a lesion as HPV related [34], ASCUS [34,35], AGCUS [36], EGD [37] and to discriminate AGCUS from neoplastic endocervical cells [38] (table 1).

**Table 1:** Cytological and histological criteria used for diagnosis of cervical lesions

Lesions	Criteria for diagnosis	Ref.
Cytologic HPV	Koilocytic atypia Multinucleation (frequently binucleation) Dyskeratosis Parakeratosis	34
ASCUS	Nuclear enlargement approximately twice the size of an immature squamous metaplastic cell Slight increase in nucleo-cytoplasmic ratio Nuclear chromatin clumping Variation in nuclear size and shape including mild hyperchromasia, binucleation, and mild irregularity in the nuclear membranes	35, 36
AGCUS	Nuclear enlargement approximately twice the size of an endocervical cell. Nuclear pseudostratification Mild hyperchromasia	37
Discriminating AGUS from neoplastic endocervical cells	Presence of normal ECCs, singly or in sheets Absence of necrosis Absence of papillary groups Mild anisonucleosis Marked chromatin distribution	37
EGD	Nuclei not cytologically malignant Nuclear hyperchromasia and pseudostratification Low mitotic figures Absence of papillary formation and cribriform pattern	38
Histologic HPV	Koilocytic atypia: 4 points Binucleation: 2 points Dyskeratosis: one point Basal cell hyperplasia: one point Papillomatosis: one point Acanthosis: one point	34

#### Colposcopic-guided biopsy and Histopathology

All EA were biopsied in an attempt to evaluate the prevalence of low/high grade dysplastic changes and invasive lesions. The received biopsies were left for 24 hours in neutral buffered formalin. Tissue embedding and processing were followed by hematoxylin and eosin (H&E) staining. The presence of bilharzial infestation was evaluated in tissue sections. Serial sections from each biopsy were classified according to the World Health Organization (WHO) for cervical neoplasia [39]. Here also strict criteria were used for histopathologic evaluation. To qualify a lesion as HPV-related, a scoring system was used for which a minimum of 6 points out of 10 were needed [34]. Data not fulfilling these criteria were classified as ASM (table 1).

#### In situ hybridization for HPVs

ISH for HPVs was effectuated for epithelial lesions starting from ASM. The materials used are summarized in table 2. Initially, HPVs were screened with the wide spectrum HPV probe recognizing genomic DNAs of HPV types 6, 11, 16, 18, 30, 31, 31, 33, 35, 45, 51 and 52. Further subtyping with HPV 6/11, 16/18 and 31/33 were performed for cases positive for pan HPV and cases negative/unsatisfactory for pan HPV but with frank HPV related lesions on conventional H&E biopsies. Positive control tissues infected with HPV were included in every ISH procedure and the technique was evaluated according to the result of this positive control. The steps for ISH are followed according to the manufacturer instructions. In brief, following deparaffinization, tissue digestion with 250–500 μl of pepsin HCl, were applied to tissue sections and the latter were incubated in 37°C oven for 10 minutes, followed by slides wash in distilled water. Denaturation and hybridization started by applying enough drops of the probe to tissue sections, coverslipping, laying the slide on pre-warmed slide warmer, and incubation in 37°C oven for 60 minutes. Slides were immersed in Tris buffer to remove covers, washed and immersed in warm stringent wash, incubated in 37°C oven for 30 minutes, and then washed in Tris buffer. For revelation, streptavidin alkaline phosphatase was applied to sections for 20 minutes. Slides were washed in Tris buffer twice. BCIP/NBT were applied to sections and incubated in dark for 60 minutes, followed by wash in Tris buffer twice. Slides were lastly washed distilled water and mounted using aqueous media. Positive signals were seen as dark blue dots in the affected nuclei.

**Table 2:** Reagents used in the study

Reagents	Source	code
*ISH detection kits and probes*		

ISH detection system	Dako	K0601
Wide spectrum HPV	Dako	Y1404
6/11 HPV	Dako	Y1411
16/18 HPV	Dako	Y1412
31/33 HPV	Dako	Y1413

*IHC detection kits and antibodies*		

LSAB-2 Detection kit	Dako	K0680
HSV- 2	Dako	B0116
CMV early antigens	Novocastra	NCL- CMV- EA
CMV late antigens	Novocastra	NCL- CMV- EA
P 24 capsid protein	Novocastra	NCL-p24

#### Immunohistochemistry

Immunohistochemistry (IHC) was effectuated for equivocal cases negative for HPV and cases with histological suspicion for infections with HSV-2 or CMV. In these instances, HSV-2, early and late CMV antigens were assessed. For other viral lesions not proven cytologically, histologically or by IHC to be HSV-2 or CMV infection, p24 capsid proteins for HIV was used. In addition, cytokeratin, leucocyte common antigen and CD79a were used to diagnose an extranodal MZBCL. The materials used are summarized in table 2. The steps of IHC are followed according to the manufacturer instructions. All incubations were performed at room temperature. Tris buffer was used for washing. In brief, following deparaffinization, the endogenous peroxidase activity was quenched by 0.3% hydrogen peroxide for 5 minutes followed by washing. Primary antibodies diluted at 1/50, were incubated for 30 minutes, then slides were rinsed in successive buffer bathes. The revelation was done with LSAB-2 detection kit. Finally, diaminobenzidine tetrachloride (DAB) was applied for 5 minutes. Slides were counterstained in Harris haematoxylin (Hx), dehydrated, cleared in xylene and coverslipped. Tissues infected with HSV-2, CMV and p24 were used as positive control while replacement of the antibodies by Tris buffer was used as negative control for the procedure. IHC results were evaluated either as positive or negative.

### Statistical analysis

Lesions were grouped according to histological results into: 1- normal without epithelial changes, 2- ASM, 3- low grade lesions including CINI with or without HPV and EGD alone or combined with either ASM or CINI, 4- High grade lesions including CINII, CINIII/CIS alone or combined with EGD and adenocarcinoma in situ (AIS), and 5- Invasive lesions. A cut-off point of 45 years was used to categorize two aged groups 35–45 and >45–60 years. SPSS version 9 was used for statistical analysis. Descriptive analysis was made of demographic, cytology and follow up CGB data. Cross tabulations with significance tests and Odd Ratio (OR) with 95% confidence interval (CI) were made. Multivariate analysis was carried out to estimate the main determinant risk factors for cacx in the study population.

### Quality control

A pilot study was carried out to test methods of obtaining community cooperation and reactions of respondents to the research procedures and their desire to participate. All staff members were trained to ensure standardization of methods. A written operational manual explaining in details how to perform Pap smear and CGB and all staining steps was prepared. Random re-screening of 20% of the Pap smear and all EA was done for every cluster, in addition to the strict criteria used for evaluating abnormal lesions and was assessed by kappa test.

## Results

### History cervical cancer and awareness of the risk factors

Twelve women (0.2%) had a female relative diagnosed as cacx. Less than 2%, mostly from urban areas, had ever had Pap smear with normal results in 77% of them. Only three women mentioned having dysplastic/atypical changes before, however these finding were not documented by cytopathological reports and their Pap smears were normal in the present study. Only 2% could identify smoking, hormones, and infection as risk factors but not HPV.

### Cytopathology

The total number of Pap smears studied was 5658. However, 5453 women with complete data were included in the study (97%). Unsatisfactory results were present in 195 smears (3%), mostly due to absence of endocervical/transformation zone and poor fixation in 66.15% and 16.92% respectively, and were more pronounced in the first three governorates. Two third of the women had inflammatory changes (63%), while epithelial abnormalities were identified among 8%.

### Inflammatory and Reparative Changes

**Figure 1:**
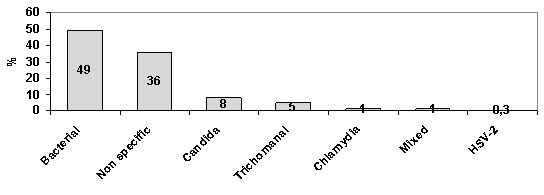
Inflammatory changes.

All the inflammatory smears were associated with reparative changes including reserve cells hyperplasia, early immature squamous metaplastic cells, cells merging to maturation and fully mature cells. This process was more pronounced in association with IUD devices. Three smears showed mature reparative changes only, probably representing post inflammatory squamous metaplasia. The most common infection was bacterial (49%) (figure 1).

### Epithelial abnormalities according to TBS

**Figure 2:**
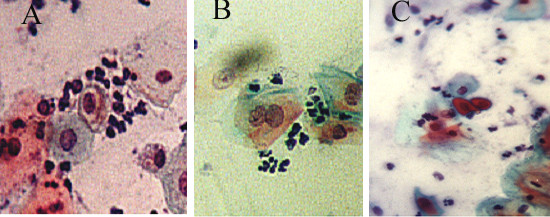
LGSIL HPV associated showing koilocytic atypia (A), binucleation (B) and dyskeratosis (C) Papanicolaou stain, × 40.

Epithelial changes were found in 424 women (8%), more commonly among LE women (9%) and less commonly in rural UE (6%); one-third of these cases were among urban governorates residents (p < 0.005). The most common type was LGSIL including HPV infection (41%) (figure 2). HGSIL was found in 22 women (5%). Cytological features highly suggestive of invasive carcinoma were diagnosed in four cases. Table 3 illustrates the EA.

**Table 3:** Percentage distribution of women by cytological epithelial abnormalities according to place of residence

Epithelial	Urban	LE		UE		P	Total	
				
abnormalities	governorates	Urban	Rural	Urban	Rural		Freq.	%
*Atypical*								
ASCUS	37.2	27.3	35.2	46.3	26.7		146	34.4
AGCUS	14.9	14.5	16.2	29.3	8.0		65	15.3
Combined	1.4	1.8	4.8	2.4	5.3		13	3.1
						<0.05		
*Squamous lesions*								
LGSIL	41.2	43.6	37.1	12.2	60.0		174	41.0
HGSIL	4.7	10.9	5.7	7.3	0.0		22	5.2
*Invasive*								
Squamous	0.0	1.8	0.0	2.4	0.0		2	0.5
Endocervical	0.7	0.0	1.0	0.0	0.0		2	0.5

Total	148	55	105	41	75		424	

### Histological evaluation of CGB

**Figure 3:**
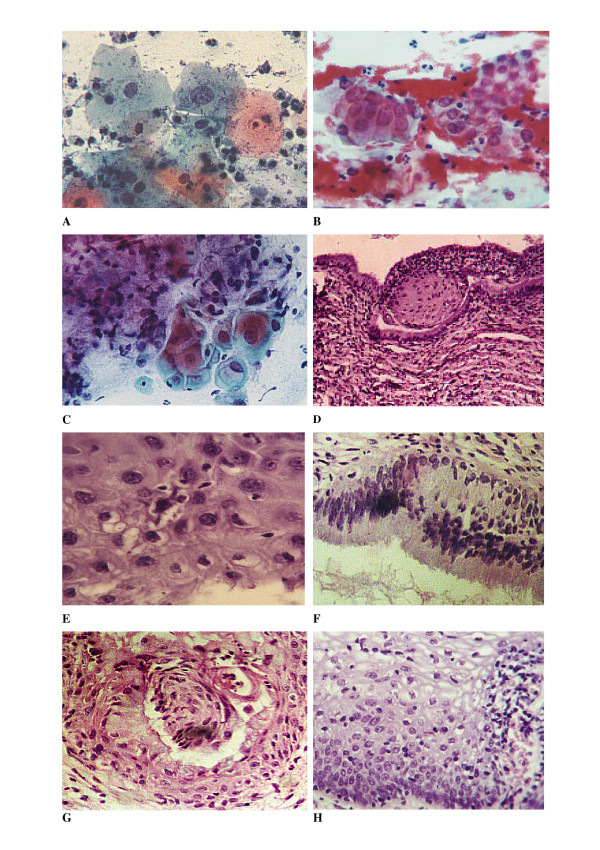
Combined dysplasia, A- ASCUS, the nucleus is twice the size of the left intermediate cell on the right, Pap stain, ×40. B- Another area showing nuclear pseudostratification and loss of the normal honey combing, Pap stain, ×40. C- A third area showing ASCUS and AGCUS in the same field, Pap stain, ×40. D- Glandular colonization by the neoplastic cells and dysplastic endocervical lining, H&E, ×10. E- Higher power magnification of the colonized glands showing koilocytes, increased nucleo-cytoplasmic ratio and large nucleolated nuclei, H&E, ×40. F- Higher power magnification of the endocervical dysplastic cells, H&E, ×40. G- Another field showing colonization of the glands by the koilocytes and residual normal endocervical cells, H&E staining, ×40. H- Surface epithelium showing koilocytic atypia, acanthosis, papillomatosis and basal cell hyperplasia, H&E, ×40.

**Figure 4:**
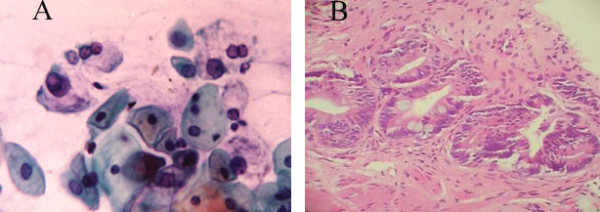
A- AGCUS favoring a neoplastic process, Pap stain, × 400. B- AIS, H&E staining, ×20.

**Figure 5:**
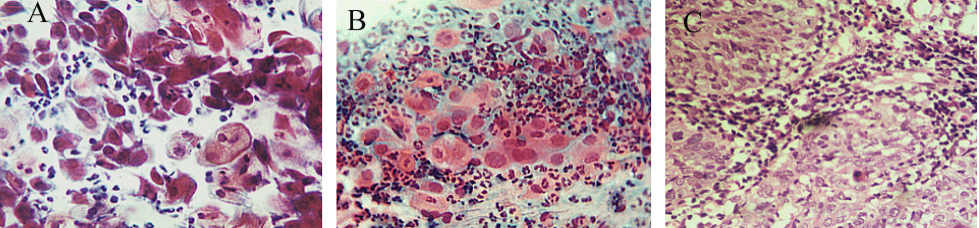
LCNK SCC, A- Fibres cells (arrow) and koilocytes (arrow head), Pap stain, × 400. B- Marked nuclear pleomorphism and hyperchromasia and heavy neutrophilic infiltrate, Pap stain, × 400. C- Corresponding biopsy: islands of neoplastic cells surrounded by heavy neutrophilic infiltrate, H&E, ×10

CGB was recommended for all women with EA (424 ones). A total of 281 ones (66%) were available. Unsatisfactory biopsies were encountered in 30 cases (10.67%); the most common cause was absence of endocervical tissue in a lesion previously diagnosed as AGCUS (62%), and was more obvious in earlier governorates. The histological results are summarized in table 4. The strict criteria used for evaluating HPVs histologically enabled us to categorize squamous lesions with or without HPV infection (table 5). However, there were no valid criteria for identification of HPVs in endocervical lesions, making ISH a premium for diagnosis of HPVs infected endocervical lesions. The overall predictive value of cervical cytology was 87% with values of 90.10%, 86.68%, 80% and 78.13% for LGSIL, ASCUS, HGSIL and AGCUS respectively. Figures 3, 4 and 5 illustrate cyto-histologic correlation of some cases.

**Table 4:** Histological results and pan HPV ISH of CGB

Results	Histology		Pan HPV ISH		
	
	Freq.	%	Negative	Positive	NS
*Normal*	32	12.8	ND	ND	ND
*ASM*	48	19.2	39	6	3

*CIN*					
CINI	111	44.4	16	89	6
CINII	11	4.4	2	9	0
CINIII/CIS	7	2.8	0	7	0

*Glandular lesions*					
EGD	12	4.8	4	8	0
AIS	2	0.8	0	1	1

*Combined dysplasia*					
ASM + EGD	4	1.6	2	2	0
CINI+EGD	18	7.2	0	17	1
CINII+EGD	2	0.8	0	2	0
CINIII+EGD	1	0.4	0	1	0

*Invasive*					
Invasive carcinoma	1	0.4	0	1	0
Lymphoma	1	0.4	ND	ND	ND

Total	250	100	63 (29.0%)	143	11
				(65.9%)	(5.1%)
			
			217 (86.8%)		

ND: not done NS: non significant

**Table 5:** Histological evidence of HPVs infection in neoplastic squamous lesions

	Freq.	Histologic evidence of HPV	%
CIN I	111	104	93.7
CINII	13	12	92.3
CINIII	7	6	85.7
Combined dysplasia	25	25	100.0
Invasive carcinoma	1	1	100.0
Total	157	148	94.3

### IHC results

Only one case cytologically diagnosed as ASCUS, was positive for HSV-2. All suspicious cases tested for CMV and HIV infections were negative by IHC. Accidentally, one case positive for leukocyte common antigen and CD79a was classified as extranodal MZBCL.

### Prevalence of HPV and cervical lesions

**Figure 6:**
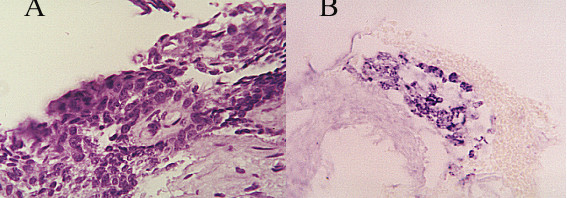
CINIII, A- H&E, × 20. B- HPV nucleic acid, ISH, pan HPV probe, × 20.

**Figure 7:**
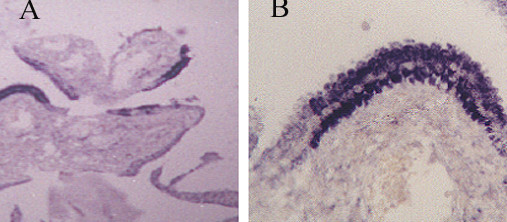
EGD, A- EGD, ISH, pan HPV probe, ×10. B- Higher power magnification showing variable degree of intensity with severe intensity in the middle zone, ×40.

**Figure 8:**
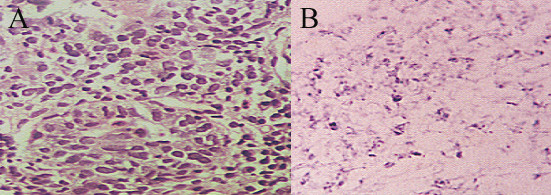
LCNK SCC A-, H&E, ×20. B- HPV nucleic acid in almost all the cells. ISH, pan HPV probe, × 20

ISH using pan HPV probe tested on 217 cases, was positive among 66%, negative in 29% and non significant in 5% of cases (figures 6, 7 and 8). The prevalence of cervical lesions in the studied population is summarized in tables 6 while that of HPV in the tested abnormal biopsies is illustrated in table 7. The viral genome for HPV 6/11, 16/18 and 31/33 was incorporated in 11.1%, 33.3% and 17.1% respectively in the studied biopsies (table 8). Interestingly, 12.5% (6/48) of ASM were HPV positive.

**Table 6:** Prevalence of cervical lesions in the Egyptian population (n = 5453)

*Cervical lesions*	Freq.	%
CINI	111	2.04
CIN I+EGG	18	0.30
CINII	13	0.24
CIN II+EGG	2	0.03
CINIII	7	0.13
CINIII + EGD	1	0.02
EGD	12	0.22
AIS	1	0.02
SCC	1	0.02
Total	166	3.04

**Table 7:** Prevalence of HPV infection in the tested population (n = 217)

*HPV infections*	Freq.	%
Pan HPV	143	65.9
HPV 6/11	24	11.1
HPV 16/18	68	31.3
HPV 31/33	37	17.1

**Table 8:** HPVs in high grade cervical lesions

	HPV				
		
	Pan	6/11	16/18	31/33	
		
High grade cervical lesions	Neg/Pos	Neg/Pos	Neg/Pos	Neg/Pos	Total
CINII	2/11	13/0	6/7	8/5	13
CIN II+EGG	0/2	2/0	0/2	2/0	2
CINIII	0/7	7/0	1/6	6/1	7
CINIII + EGD	0/1	1/0	0/1	0/1	1
SCC	0/1	1/0	0/1	1/0	1
AIS	1/0	1/0	1/0	1/0	1
Total	3/22	25/0	8/17	18/7	25

### Risk factors of cervical lesions

Risk factors of ASM were infection with HPV especially subtypes 16/18 and 31/33 and ever usage of vaginal contraceptives (p <0.0001 for each). The risk factors for low grade CIN are still menstruating (p <0.001), working (p <0.0001), HPV infection with the three probes studied 6/11, 16/18 and 31/33 (p <0.000 for each), while for high grade CIN infection, with HPV mainly subtypes 16/18 and 31/33 (p <0.001) was the common threat. Multiple regression analysis revealed that being a working woman (OR 2.0, 95% CI 1.3–3.1) and still menstruating (OR 1.5, 95% CI 1.1–2.1) were the main risk factors.

HPVs infection were more pronounced in younger age, actually married, still menstruating and ever used hormonal or vaginal contraceptives (p <0.05 for each), and unskilled workers (p <0.0001). In addition, there was an increasing positivity rate for HPV with increasing lesion severity. Applying multiple regression analysis revealed that being a working woman (OR 2.0, 95% CI 1.3–3.2), still menstruating (OR 1.6, 95% CI 1.1–2.3) and women who ever had hormonal contraception (OR 1.5, 95% CI 1.1–2.1) were the main determinants for HPV infection. HPV infection seemed to be more among women whose husbands ever had another wife or travelled before, however this finding was not statistically significant.

## Discussion

HPV has been recognized as the necessary cause of cervical cancer [2]. However, in the present study, awareness of HPV as major risk factor for cacx was very low among the studied population while smoking, hormones, and infections were identified as main risk factors. Previous studies approaching this point came out with the same conclusion of ignorance of association between HPV and cacx [40,41]. As expected, only 1.5% of studied women, mostly from urban areas, had Pap smear. Three of them mentioned, without documents, having results consistent with invasive lesions. However, their cytological results were normal in this study. This important point highlights the absence of health culture in the population. Studies from other countries, reported that only 5% of women in low and middle income countries received screening, usually in private clinics and few urban settings, contrary to high-income countries where up to 70% of women were screened [1,42].

ASM cases were positive for HPV 16/18 and 31/33. ASM, a poorly characterized cervical lesion with uncertain biological and clinical significance, shares some but not all morphological features of SIL [43,44]. It has been shown that HPV positive ASM biopsies were significantly more likely to have concurrent or subsequent diagnosis of HSIL than HPV negative ones [43,45]. Unfortunately, the actual study is limited by the fact that it covers a single period and had no opportunity to follow up ASM HPV positive cases. The rational for inclusion of ASM was based on the fact that recent studies found HPVs genome incorporated in normal squamous epithelium [22,46,47]. However, due to financial restraints, normal tissues were not included, to evaluate the presence/absence of HPVs.

This community based study confirms previous hospital based one reporting low prevalence rate for CIN; analysis of 4458 patients showed a prevalence of 0.36%, 0.23% and 0.12% for CINI, CINII and CINII respectively [48]. In addition, the Middle East Cancer Consortium reported 0.027% prevalence rate for cacx for Egyptian women [49]. The prevalence of EA and HPV infections in Egypt is comparable to other geographical areas in Muslim and Middle East countries. In Saudi Arabia, EA represented 3.14% out of 3088 screened women with ASCUS, LGSIL, HGSIL, invasive SCC, AGCUS and adenocarcinoma represented 0.45%, 0.93%, 0.55%, 0.13%, 0.13% and 0.03% respectively [50]. In Jordan a prevalence of 0.026% has been reported [49]. In Lebanon, a study conducted on 1,026 women revealed a prevalence of 4.9% for HPV with type 16 representing 3% [51]. In Morocco, 70.5% of invasive carcinoma cases were HPV positive, 34.88% cases had HPV16, and 15.5% cases had HPV18 [52]. In Iran, controversial data were reported; 73.9% of HPV positive cacx contained HPV16 [53], while in another study HPV16 was identified in 26.7% and none of the samples were positive for HPV18 [54].

Studies showed significant geographic variation in the prevalence of oncogenic viral types in cervical lesions with roughly half of all cacx worldwide containing HPV 16. Other important high risk types are HPV 18, 45, and 31. Less prevalent high risk types include HPV 26, 33, 35, 39, 51, 52, 56, 58, 59, 68, 73, and W13b [47]. In Africa, the most common subtype is HPV 16 followed by 45, 18, 31 and 33 [55], a point concordant with the actual work. Unskilled workers were at greater risk for HPV infection and other EA. These women were the highest to have husbands with history of genital infection, showed vaginal discharge by examination, reported history of infection and higher sexual activity. HPV infection seemed to be more among women whose husbands ever had another wife or travelled before. In addition, one fourth of unskilled workers had husbands who ever travelled before or married another wife. In spite that the majority of women had married once, 15% of unskilled workers had married more than once. It is clear that the marital/sexual practices of unskilled workers were the determinants of their higher susceptibility to HPV and CIN. The socio-economic characteristics of unskilled workers confirm previous studies where associations have been found between low socioeconomic status [1,28,26], multiple sexual partners [56-59] and cacx. Another risk factor, smoking, is added to unskilled workers, as 83% of them were exposed to smoking at home and work. Higher prevalence for high-risk HPV types was significantly associated with cigarette smokers [60]. Steroid contraception has been postulated to be one mechanism whereby HPV exerts its oncogenic effect on cervical tissue especially among long-term users [61], a point concordant with the present study, mostly noted in one half of unskilled workers. The prevalence of HPV in ASM and other abnormal biopsies may be underestimated due to the fact that the HPV probes used in the study do not cover all the high risk types and include some low risk [47]. Additionally, normal tissues previously shown to harbour the virus [22,46,47] were not tested due to financial limitations. A third important point is the use of ISH technology, an insensitive one compared to catalyzed signal amplified colorimetric DNA ISH [62], to hybrid capture (HC) HPV DNA assay [63-65] or polymerase chain reaction [66,67]. However, other studies showed that ISH HPV was more predictive of biopsy histopathology in patients with detectable cervical lesions than is HC HPV [68]. Furthermore, the sensitivity of ISH was comparable to that of HC2, with significantly superior specificity, and was therefore thought to be an efficacious alternative to HC2 for triaging patients with abnormal cervical cytology results [69].

Our data on schistosomiaisis as risk factor for the development of cacx confirm previous studies in Egypt [8,10] and elsewhere [25]. However, T. vaginalis previously implicated to have direct relation to cacx in the Egyptian population [11] were not established in the actual work. Moreover, early marriage or early sexual relations, was not significantly associated with HPV as Egyptian women start sexual relations with marriage. Other studies confirmed that women who have their first intercourse at an early age are at high risk for HPV infection and cacx [56,57,70]. Being uncircumcised male was found to be a high risk factor [59], which was not evaluated due to universality of male circumcision among Egyptian men both Moslem and Christians.

The study is limited by the fact that it covers a single period and had no opportunity to examine trends over time. Three obstacles reduced the number of satisfactory smears, noted mostly at the beginning of the study, implying the importance of training. These were, smears not interesting the squamo-columnar junction, inadequate fixation and hemorrhagic smears resulting from traumatisation of the mucosa by the cytobrush. Inadequate biopsies were encountered whenever they were performed in health units using portable colposcope. In addition, women of higher socioeconomic group refusal to participate may have led to missing the chance to describe the pattern of cervical lesions among this group. The drop out of women for CGB was due to their refusal to participate in spite of counselling. Concerning HPVs ISH, two points should be discussed. The first was fixation more than 24 hours in UE specimens, a point that may explain the negativity for all HPVs probes even with the presence of frank HPVs in biopsies. The second was the limited number of tested probes compared to the known HPVs subtypes. Cultural sensitivity for sexual relationships in the Egyptian community in general and among women specifically, made it difficult to get detailed information even with using probing questions for assessing sexually transmitted infections.

In conclusion, this study is the first one at a national level describing the characteristics of women with cervical epithelial abnormalities. They were mostly of middle income, married, with three and more children, mostly uneducated and not working. The awareness of the Egyptian women by risk factors or symptoms of different reproductive health problems are extremely low. Prevalence of CIN and invasive lesions was 3.1% and 0.04%, while the prevalence of HPVs was 2.6% and was positive in 94.3% of cervical lesions confirming that it is the main causing agent. The study recommends introducing cacx screening for all women at least once every 10 years for women with normal cytological findings, and yearly for three successive years for inflammatory changes. CGB should be performed to all EA to avoid losing patients and subsequently tumor progression. In addition, raising awareness of Egyptian women on reproductive health and risk factors of cacx through specially designed health communication programs is mandatory.

## Abbreviations

AGCUS: atypical glandular cells of undetermined significance, AIS: adenocarcinoma in situ, ASCUS: atypical squamous cells of undetermined significance, ASM: atypical squamous metaplasia, Cacx: cancer cervix, Chlamydia T: Chlamydia trachomatis, CIN: cervical intraepithelial neoplasia, EA: epithelial abnormalities, EGD: endocervical glandular dysplasia, HGSIL: high grade squamous intraepithelial lesion, HPVs: human papilloma viruses, IHC: Immunohistochemistry. ISH: in situ hybridization, LGSIL: low grade squamous intraepithelial lesion, Pap smears: Papanicolaou smears, TBS: the Bethesda System, SCC: squamous cell carcinoma.

## Authors' contributions

HA was the Principal Investigator for Pathology, AR was the Principal Investigator for Data Management and KD was the Principal Investigator for Field Work.
